# Mechanical performance of negative-stiffness multistable bi-material composites

**DOI:** 10.1007/s00707-024-04158-9

**Published:** 2024-12-23

**Authors:** Navid Mehreganian, Shayan Razi, Arash S. Fallah, Pooya Sareh

**Affiliations:** 1 Creative Design Engineering Lab (Cdel), School of Engineering, University of Liverpool, Liverpool, L69 3GH UK; 2 Department of Civil and Environmental Engineering, Stanford University, Stanford, CA 94305 USA; 3 Department of Mechanical, Electronic, and Chemical Engineering, OsloMet, Pilestredet 35, St. Olavs Plass, 0166 Oslo, Norway; 4 Creative Design Engineering Lab (Cdel), School of Engineering, Newcastle University, Newcastle upon Tyne, NE1 7RU UK

## Abstract

Architected latticed structural systems, known as metamaterials or metastructures, have recently garnered significant attention due to their superior performance under various loading conditions. This class includes metamaterials exhibiting multistability, characterized by negative stiffness, which enables energy entrapment during transitions between equilibrium states, making them suitable for applications such as lightweight protective systems. In this study, in three folds, we investigate the mechanical performance of a negative stiffness honeycomb metamaterial (NSHM) with unit cells composed of curved double beams. First, the quasi-static compressive response is numerically examined using the finite element method, revealing that this response is independent of the number of cells. Next, we analyze the transient dynamic response of both mono-material NSHMs and bi-material composites, where the stiffeners are replaced by brittle polystyrene, under localized striker and uniform plate impacts. Finally, we present an analytical model for the total potential energy, with solutions obtained through an optimization technique, and validate these results against the numerical simulations. Through these analyses, we study the effects of several parameters influencing multistability. Our findings demonstrate that the bistability ratio significantly impacts the overall response of the honeycomb, and the desired negative stiffness can be achieved with high bistability ratios. Additionally, the contact force peaks resulting from striker impact are found to be independent of the number of constituent elements. The optimized geometry of the lattice is determined through a trade-off between porosity and stiffness, achieved by thicker cell walls.

## Introduction

The advent of additive manufacturing has given rise to significant technological advancements such as the design and fabrication of structured materials at different length scales. Additive manufacturing by means of 3D printing has, as such, made the fabrication of several types of novel mechanical metamaterials and metastructures of versatile shapes and sizes possible. Metamaterials are a class of materials the internal microstructure of which is engineered to manifest peculiar properties, such as wave manipulability, negative refractive index, chirality, axial-distortional coupling, to name but a few. Metastructures are similarly designed, however, in contradistinction to metamaterials, the ratio of unit cell dimensions in the tessellation to overall structural dimensions in this case is not infinitesimal. These structural classes possess properties that may not be readily found in natural materials, making them attractive for specific applications.

Perhaps, following the invention of negative refractive index optical metamaterials by Pendry [1], harvesting such distinctive, yet desirable, properties has attracted the keen interest of researchers with applications in various fields, including optics [2–11], phononics [12–24], shape-shifting structures [25–39], and impact engineering [40–45].

The fascinating properties of these materials arise not from the base material itself but from their microstructural arrangements. Their unique constitutive behavior is a result of their morphology, topology, and the scale at which they are fabricated. Despite variations, they share a common feature: they are constructed as periodic (or in some cases, regular non-periodic [46]) lattices of a constituent unit cell. This design approach imparts desirable properties, such as negative stiffness (in multistable metastructures), negative effective mass density and bulk modulus (in phononic metamaterials), or negative Poisson’s ratio (in auxetic metamaterials).

**Fig. 1:**
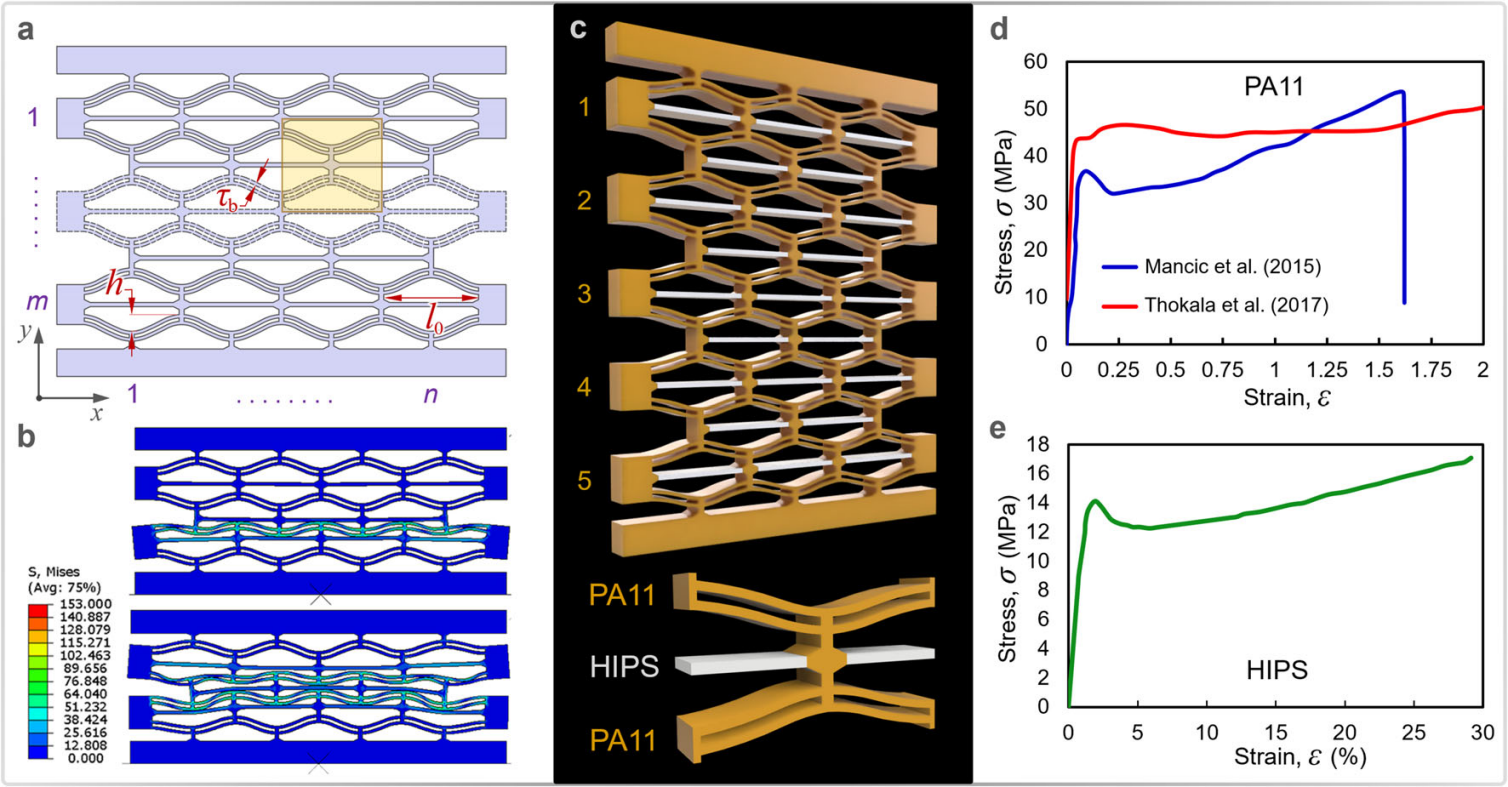
**a** Schematics of an $m\times n$ arrangement of curved double beams (CDBs). **b** Finite element simulation of the response of a typical NSHM lattice under a static compressive load. **c** A composite bi-material lattice (top) and its constituent element (bottom). **d** Stress–strain curves of Polyamide 11 (PA11) (adapted from [52, 53]). **e** Stress–strain curves of high impact polystyrene (HIPS) (adapted from [54])

Negative stiffness metastructures correspond to bistable or multistable systems that exhibit transitions from one stable equilibrium state to another. An interesting example of multistable, tunable metastructures is the recently fabricated negative stiffness honeycomb metastructure (NSHM), which when subjected to lateral unidirectional loads, exhibit close to zero Poisson’s ratio and negative stiffness as its most conspicuous features [47, 48] (see Fig. 1a–c). The negative stiffness refers to a decrease in the force magnitude following a jump in the displacement field of the honeycomb structure. This phenomenon enables the architected metastructure to serve as an elastic energy-entrapping component. Such a mechanism depends solely on reversible changes in the state of prescribed geometries, rendering the response independent of scale, deformation rate, and loading history [49]. According to some previous studies [50, 51], the fabricated negative stiffness honeycomb structure exhibited higher recoverability (i.e., negligible plastic deformation) compared to its conventional hexagonal honeycomb counterparts.

Following the work of Qiu et al. [55], the negative stiffness lattice structure composed of individual or paired curved beams has been examined with various geometrical modifications, as noted in [56, 57]. These studies assessed the instability of a mechanical system’s representative volume element (RVE), consisting of two or more vertically paired curved beams, where the top beam was thinner than the bottom one. The hysteresis map of the structure under both displacement-control and load-control conditions was highlighted and elucidated. It was observed that the curvature of the top thin beam changed only when the edges of the paired beams were stretched. In a lattice structure made of such an RVE, where the middle beams were shorter than the top and bottom ones, curvature reversal was observed in both the shorter middle beams and the top thicker, longer beam, while the shape of the bottom beam remained unchanged across various loading scenarios. In a similar study [58], the snap-back phenomenon was observed in thick hyperelastic columns, where both force and displacement levels decreased after the onset of loading. Ren et al. [59] investigated the mechanical performance of a three-dimensional bistable negative stiffness metamaterial composed of curved beams and diagonal bracing. The response of the periodic structure was characterized by a parabolic path of the average force in the force–time history curves, with multiple fluctuations around the average force magnitude before settling off to zero. The relief of the structure from the impact force was manifested as residual vibrations in the force–time history around the zero-force line.

Zhakatayev et al. [48] modified the model presented in [55] and developed an analytical model for the NSHM composed of curved beam pairs. Goldsberry and Haberman [60] examined the tunability and bandgaps of the NSHM lattice subjected to axial strains. A finite element model was utilized to simulate the static deformation and the subsequent linear wave motion in the pre-strained state. Their findings indicated that the NSHM exhibited significant tunability and a high degree of anisotropy, which could be used to direct wave energy based on static pre-strain levels. Additionally, it was demonstrated that partial band gaps existed, where only longitudinal waves propagated.

From a load–displacement perspective, the snap-through instability phenomenon is characterized by a sudden jump in displacement on the load-control curve. A counterpart phenomenon, known as snap-back, occurs on the displacement-control curve when the load decreases suddenly, even without an increase in the prescribed displacement [57]. During snap-back, the profile of the curved beam reverses from bulging downward to bulging upward. In a lattice composed of multiple arrangements of bistable beams in a modular pattern, the force landscape of the snap-buckling chain mechanisms exhibits erratic curves, shifting between various equilibrium states, leading to the evolution of energy entrapment. Consequently, the lattice exhibits hysteresis during loading and unloading cycles.

The dissemination of this paper is organized into six sections. Following the introduction of Sect. 1, Sect. 2 develops the fundamental mathematical expressions for the response of the bistable curved beam. Section 3 examines the phase-transforming features of the NSHM lattice, composed of varying numbers of cells, under both static and dynamic striker impact loading conditions. Section 4 investigates the dynamic response of the composite lattice subjected to plate impact. In Sect. 5, we develop an optimization algorithm to evaluate the transverse deformation of the curved double beams (CDBs) and the strain energy of the lattice, which is then validated against numerical finite element (FE) models. This section also includes a discussion on the morphology optimization of the microstructure. Finally, Sect. 6 provides the concluding remarks of the study.

## Governing equations

In this section, we examine the mechanics of a multistable NSHM model with geometry similar to those examined in [47, 60–62]. A constituent element of such a model is illustrated in Fig. 1a and c. The morphology of the NSHM consists of an *m* × *n* periodic lattice of the constituent elements with *n* cells in the horizontal direction and *m* cells in the vertical direction (equivalent to the number of layers), as shown in Fig. 1a and c. The constituent elements in this structural arrangement are identified top-down using the matrix notation ${\text{C}}_{mn}$, while the (horizontal) struts confined between the two pairs of curved double beams (CDBs) in cell ${\text{C}}_{mn}$ are referred to as ${\text{Ch}}_{mn}$. Each constituent element consists of two pairs of CDBs made of Polyamide 11 (PA11), which are interconnected with vertical and horizontal stiffeners.

**Fig. 2:**
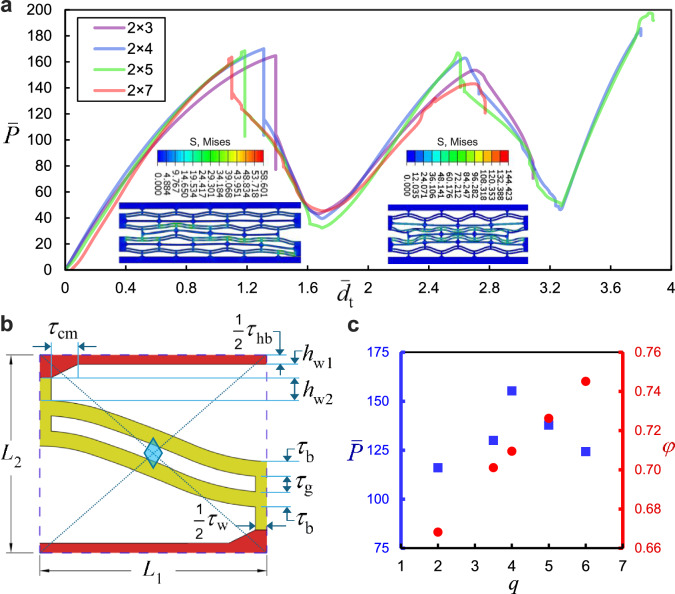
**a** Force–displacement convergence of the homogenized microstructure for $q=4$. **b** Geometry of the representative unit with two-fold symmetry. **c** Influence of the bistability ratio on the porosity and peak force of the microstructure

These stiffeners provide shear stiffness, i.e., resistance to shear strains, and help restrict the rotational degrees of freedom (DOFs). Depending on the application of the NSHM, the composite may be classified as a mono-material one, such as one made entirely from PA11 [61], or a multi-material composite, where the stiffeners are fabricated from a material of distinguishably different stiffness. Recently, Liu et al. [63, 64] investigated the energy absorption of a bi-material NSHM, wherein the rigid PLA core comprising the cell walls and stiffeners was enclosed by a soft outer skin incorporating the CDBs made of thermoplastic polyurethane (TPU). The initial geometric profile is given as follows

1 $${w}_{0}=h{\text{sin}}^{2}\left(\frac{\pi x}{{l}_{0}}\right)$$

where ${l}_{0}$ and $h$ denote the span and apex height of the beam, respectively. The curved beams are paired with a central strut of width ${\tau }_{\text{w}}$ and gap distance of ${\tau }_{\text{g}}$, forming a CDB. These CDBs have a sinusoidal profile which mimics the flat beam deformed into its first Euler buckling mode, as given in Eq. 1, similar to those in [55, 62]. Using the Voronoi decomposition, the Wigner–Seitz unit cell of the lattice can be generated and constructed by perpendicular bisectors. The quarter symmetry of the constituent element, equivalent to the first Brillouin zone in the reciprocal lattice, is illustrated in Fig. 2b. The aperture area of this representative unit is given as

2 $$A_{{\text{a}}} = 2\int\limits_{0}^{{l_{0} /2}} {w_{0} dx + h_{{\text{w}}} l_{0} - h_{{{\text{w}}1}} \tau _{{{\text{cm}}}} + \frac{1}{2}\tau _{g} (s_{0} - \tau _{w} )}$$

The infill area of this representative unit is expressed as

3 $${A}_{\text{f}}={\tau }_{\text{b}}({s}_{0}-{\tau }_{w})+{\tau }_{\text{w}}\left({h}_{\text{w}}+{\tau }_{\text{g}}+2{\tau }_{\text{b}}+\frac{{\tau }_{\text{hb}}}{2}\right)+\frac{{\tau }_{\text{hb}}{l}_{0}}{2}+{h}_{\text{w}1}{\tau }_{\text{cm}}$$

**Fig. 3:**
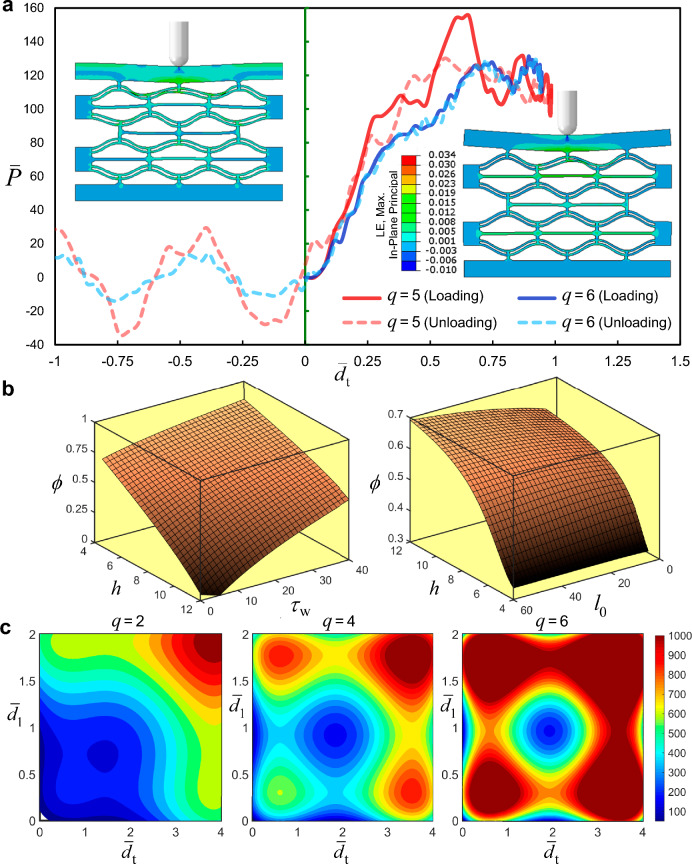
**a** Force–displacement hysteresis of lattice with $q=5$ and $q=6$ due to the striker impact. **b** Interaction surface of the lattice geometric parameters ($h,{l}_{0},{\tau }_{\text{w}}$) with porosity. **d** Contour plots of the strain energy density for different values of $q$

The total area of the representative unit is ${L}_{1}{L}_{2}={A}_{\mathrm{f}}+{A}_{\mathrm{a}}$. The porosity of the representative unit is thus given as $\phi ={A}_{\mathrm{a}}/({L}_{1}{L}_{2})$, and the relative density is calculated as $\widehat{\rho }={A}_{\mathrm{f}}/({L}_{1}{L}_{2})$. The interaction surface of the beam apex height, initial length, and cell wall thickness is presented in Fig. 3b and c.

For convenience, the parameters characterizing the transverse deformation of the NSHM lattice are nondimensionalized, using the Buckingham π theorem, as follows:

4a–g $$\overline{U }=\frac{U{l}_{0}^{3}}{EI{h}^{2}}, \quad {\overline{w} }_{j}=\frac{{w}_{j}}{h}, \quad q=\frac{h}{{\tau }_{\text{b}}}, \quad \overline{{{P}}}=\frac{P{l}_{0}^{3}}{EIh}, \quad \overline{p }=\frac{p{l}_{0}^{2}}{EI}, \quad \overline{G }=\frac{G}{m}, \quad \overline{V }=\frac{{V}_{0}}{h}$$

where $\overline{U }$ represents the energy parameter, $w$ is the displacement of the curved beam, $p$ is the membrane force, ${l}_{0}$ is the initial length of the beam, and $q$ is the bistability ratio, which is a dimensionless parameter that may be used as a measure of the CDB stiffness to exhibit bistability/metastability, $G$ is the striker’s mass and ${V}_{0}$ is the striker’s initial velocity, $P$ represents the actuation force, while $E$ and $I$ are the Young’s modulus and the second moment of area of the beams, respectively. The displacement profile of the curved beam for the ${j}^{\text{th}}$ mode is given as ${\overline{w} }_{j}={A}_{j}{W}_{j}(x),$ where

5a $${W}_{j}=1-\text{cos}\left({\omega }_{j}X\right),\hspace{0.4cm}\text{ for } j=\text{1,3},5,\dots .$$

5b $${W}_{j}=1-2X-\text{cos}\left({\omega }_{j}X\right)+\frac{2\text{sin}\left({\omega }_{j}X\right)}{{\omega }_{j}}, \hspace{0.4cm} \text{ for } j=\text{2,4},6,\dots .$$

with ${\omega }_{j}=( j+1)\pi$, $X = x/{l}_{0}$, and ${\overline{w} }_{j}={A}_{j}{W}_{j}$. The displacement parameter ${\overline{w} }_{j}$ is the dimensionless form of the transverse modal displacements of the ${j}^{\text{th}}$ mode, and ${A}_{j}$ and ${W}_{j}$ correspond to the generalized coordinates and mode shapes (in dynamics, these represent the temporal and spatial components of the modal displacement), respectively.

The mode shapes are outlined in Eq. 5, for odd and even indexed terms, respectively, then using the mode superposition method, the displacement profile of the beam can be expressed as a linear combination of the mode shapes weighted by the generalized coordinates as amplitudes ${A}_{j}$, such that the total dimensionless displacement of a curved beam is given as $\overline{w }={\sum }_{i=1}^{\infty }{\overline{w} }_{i}$ where $\overline{w }$ is the curved beam displacement per its apex height. The total potential energy ${U}_{\text{t}}={U}_{\text{b}}+{U}_{\text{m}}-W$ of the system can now be formulated as the algebraic sum of the total strain energies pertaining to each CDB pair for the structure subtracting the work done $W$ on it. In formulating the total potential energy, of the system, the stiffeners are assumed as rigid components with no deformability and thus no contribution to strain energy or work, i.e. of zero potential energy.

It should be noted that, while all modes may contribute to the strain energy of the CDBs and in turn, the periodic lattice, only certain modes contributing to the mid-point displacement $d$ of the beam affect the transverse displacement of the lattice. When loaded transversely, the beam pairs in which the second mode is constrained, provided the apex height-to-beam thickness ${\tau }_{\text{b}}$ ratio $q$ is sufficiently large ($q=h/{\tau }_{\text{b}}>2.31$) they can exhibit multistability, i.e. they undergo multiple stable configurations that permit the CDBs to go through transition from mode 1 to 3 (and/or higher modes) without undergoing mode 2 deformation. In the force–displacement landscape of the lattice, this is manifested by a series of snap-through and snap-back jumps, where the former renders possible a jump in the displacement without any changes in the applied force magnitude, while the latter precipitates a sudden reduction of the force level with little or no change in the displacement field. Due to the presence of both inverted and upright CDBs, the two buckling phenomena are interspersed. It is, however, shown in the sequel that under certain conditions, the lattices with a smaller number of apex height (e.g., $q=2$) can manifest limited negative stiffness features when loaded transversely.

As the beams in the CDBs are paired by a central strut (also referred to as a center plunger, e.g., by [55]), their mid-point displacements are synchronous. For the sake of simplicity, in the theoretical analyses of this section, it is further assumed that the remaining portions of the curved beams deform transversely in harmony and there exists insignificant or zero relative deformation between them. This assumption is validated by the analyses in the sequel. While a relative deformation of the beams in the CDB pair may arise from the presence of mode 2 or higher modes in them, only a certain number of modal deformations ${A}_{j}$ contribute to the mid-point transverse displacements of the CDBs as ${\overline{d} }_{i}=1-2{\sum }_{j=\text{1,5},9,\dots }{A}_{j}$ (with $\overline{d }=d/h$), where ${\overline{d} }_{i}$ is the dimensionless displacement of the ${i}^{\text{th}}$ beam. Clearly, ${\overline{d} }_{i}$ may arise due to both the snap-buckling of the upright CDBs and the snap-back of the inverted CDBs, however, the order in which this transpires depends on the type of loading. Concerning the time-independent scenarios such as the static lateral loading, following the full buckling of the upper CDBs of each constituent element, the stiffness of such a beam pair is assumed to be lost. Consequently, the strain energies of these beams are removed from the Lagrangian functional of the lattice, while energy transfer to the lower portion of the cell induces snap-buckling of the lower CDBs at the same level.

The initial length of the beam in the dimensionless form ($${S}_{0}={s}_{0}/{l}_{0})$$ reads:

6 $${S}_{0}=1+\frac{{h}^{2}{\omega }_{1}^{2}}{16{l}_{0}^{2}}$$

where ${\omega }_{j}=( j+1)\pi$. The dimensionless membrane force, $\overline{p }$, is given as

7 $$\bar{p} = \frac{{3q^{2} }}{{4S_{0} }}\left( {\omega _{1}^{2} - 4\sum\limits_{{i = 1}}^{\infty } {A_{i}^{2} \omega _{i}^{2} } } \right)$$

Hence, it transpires that the bending ${\overline{U} }_{\text{b}}$ and membrane ${\overline{U} }_{\text{m}}$ strain energies of each curved beam are expressed, respectively, as

8a–b $$\bar{U}_{{\text{b}}} = \frac{{\omega _{1}^{4} }}{4}\left( {\frac{1}{2} - A_{1} } \right)^{2} + \frac{1}{4}\sum\limits_{{i = 2}}^{\infty } {A_{i}^{2} \omega _{i}^{4} {\hspace{0.25cm}\text{ and }}\hspace{0.25cm} \bar{U}_{{\text{m}}} = - \frac{{\bar{p}}}{4}\sum\limits_{{i = 1}}^{\infty } {A_{i}^{2} \omega _{i}^{2} } }$$

Assuming that initially the CDBs are stress-free, the total potential energy of ${i}^{\text{th}}$ curved beam is given as

9 $$\bar{U}_{i} = \frac{1}{4}\left( {\omega _{1}^{4} - \bar{p}\omega _{1}^{2} } \right)\sum\limits_{{j = 1}}^{n} {A_{{j,i}}^{2} \left( t \right) - \bar{P}\left( {1 - 2\sum\limits_{{j = 1,5,9,13,...}}^{\infty} {A_{{j,i}} \left( t \right) - 4\pi ^{4} A_{{1,i}} \left( t \right) + 4\pi ^{4} } } \right)}$$

where $t$ in ${A}_{j,i}(t)$ denotes the time variable. The modal amplitudes can be derived to minimize the total potential energy of the structure, ${\overline{U} }_{\text{t}}$. To this end, the extremum condition of the total potential function dictates that its first variation must be zero; in other words, the derivative of ${U}_{\text{t}}$ with respect to each modal amplitude must vanish, i.e., $\partial {\overline{U} }_{\text{t}}/\partial {A}_{j}=0$. It thus follows that the three expressions derived can fully characterize the force–displacement relationship of a curved beam, indicating that either mode 2 or mode 3 should be constrained. In practical applications, only the second mode can be constrained, either by connecting a central plunger or, more conveniently, by pairing and clamping the curved beams at the center. The constraint imposed by the central clamp on the rotational degree of freedom translates the transverse displacement into axial deformations in the beams on either side of the center [55]. In any case, the presence of mode 2 has negligible influence on the overall profile of the force–displacement curves of the NSHM. Therefore, using Eqs. 9 and 14, the two different representations of the solution for force $\overline{P }$ are given as follows in Eq. (10a-b):

10a $$\overline{P }=\frac{{\pi }^{4}\overline{d}}{2{S }_{0}}\left[3{q}^{2}\left({\overline{d} }^{2}-3\overline{d }+2\right)+4+\frac{{\pi }^{2}{h}^{2}}{{l}_{0}^{2}}\right]$$

or

10b $$\overline{P }=\frac{1}{4}\left({\overline{p}A }_{1}{\omega }_{1}^{2}+\left(\frac{1}{2}-{A}_{1}\right){\omega }_{1}^{4}\right),$$

which has two turning points as

11 $${\Delta }_{1},{\Delta }_{2}=1\mp \frac{1}{3}\sqrt{3-{\pi }^{2}{\tau }_{\text{b}}^{2}{l}_{0}^{-2}-{q}^{-2}} ,$$

12 $$h\ge \frac{2{l}_{0}{\tau }_{\text{b}}}{\sqrt{3{l}_{0}^{2}-{\pi }^{2}{\tau }_{\text{b}}^{2}}}$$

with ${\Delta }_{2}+{\Delta }_{1}=2$. Furthermore, for the beam to experience snap-through, Eq. 11 dictates that ${l}_{0}>\pi {\tau }_{\text{b}}/\sqrt{3}$, in addition to the apex height assuming values of:

which can be simplified to $q\ge (2/\sqrt{3}) (1-{\pi }^{2}/6{{l}_{0}}^{2})$.

## Mono-material NSHM composite

### Static compressive loading

Numerical finite element (FE) models were set up in the software ABAQUS/Explicit to investigate the mechanical response of the lattice with an arbitrary number of constituent elements. This study supplements previous research [61] and explores the mechanics of bi-material NSHM composites, particularly the influence of horizontal components on the overall lattice response.

The apex height of the curved beams varied as $h=2, 3.5, 4, 5, \text{and }6 \text{ mm}$, consistent with the authors’ previous studies [61, 62], with the initial beam span and curve beam thickness being ${l}_{0}=60 \text{ mm}$ and ${\tau }_{b}=2 \text{ mm}$, respectively (for a complete list of lattice dimensions, the reader is referred to [61]). The lattices were presumed two-dimensional and discretized with CPS4R plane stress quadrilateral, reduced integration, and hourglass controlled elements with an average mesh size of 0.5 mm. The out-of-plane thickness of the models, denoted as *b*, was assumed to be 15 mm. The shells were made of PA11, with a Young’s modulus of 1582 MPa, a Poisson’s ratio of 0.33, and a density of 1.04 g/cm^3^.

The plastic flow and damage in the elements of the composite lattice will be discussed in Sect. 4. The CDBs are connected by vertical struts and horizontal stiffener beams. The latter provide shear resistance and cause the CDB pairs to be tied on either side. This configuration eliminates lateral deformations, rendering the overall deformation homogeneous. The lattice was fixed at the bottom end, while transverse loading was applied at the top using a displacement-control condition, with a velocity of 0.01 mm/s for a duration of 3200 s. A periodic boundary condition (PBC) was applied to the top layer to ensure continuity of stress and displacement, as outlined in [65–67].

The PBC can be used to homogenize data from a 3D microstructure to a meso- or macro-structure, provided the assumption of periodicity holds. The difference in displacement components across a pair of parallel opposite ${k}^{\text{th}}$ boundary surfaces are given as ${u}_{i}^{(k+)}-{u}_{i}^{\left(k-\right)}={\varepsilon }_{ij}^{0}({x}_{j}^{\left(k+\right)}-{x}_{j}^{\left(k-\right)})={\varepsilon }_{ij}^{0}\Delta {x}_{j}^{k}$, where indices *k* + and *k* − identify the ${k}^{\text{th}}$ pair of the two opposite parallel boundary surfaces of the representative unit. Each point ${x}_{j}^{(k+)}$ on the boundary $\delta {\Omega }^{+}$ of the representative unit is associated with a unique point ${x}_{j}^{(k-)}$ on the opposing part $\delta {\Omega }^{-}$ of the representative unit [65, 66]. The geometric model analysed for the optimization and validation of the theoretical model is a variation of the models examined in [47, 61, 62] shown in Fig. 1. It incorporates a thick center plunger width of ${\tau }_{\text{w}}=11\text{ mm}$ to mitigate mode 2 deformations, similar to the model examined in [68] with a thick center plunger, except that the curved beams in this model are paired. Several other models were investigated to optimize the metastructure through altering the gap displacement and/or increasing the thickness of the center plunger to enhance the modal synchronization of the beams in the CDB pair. However, achieving modal synchronization is nearly impossible.

**Fig. 4:**
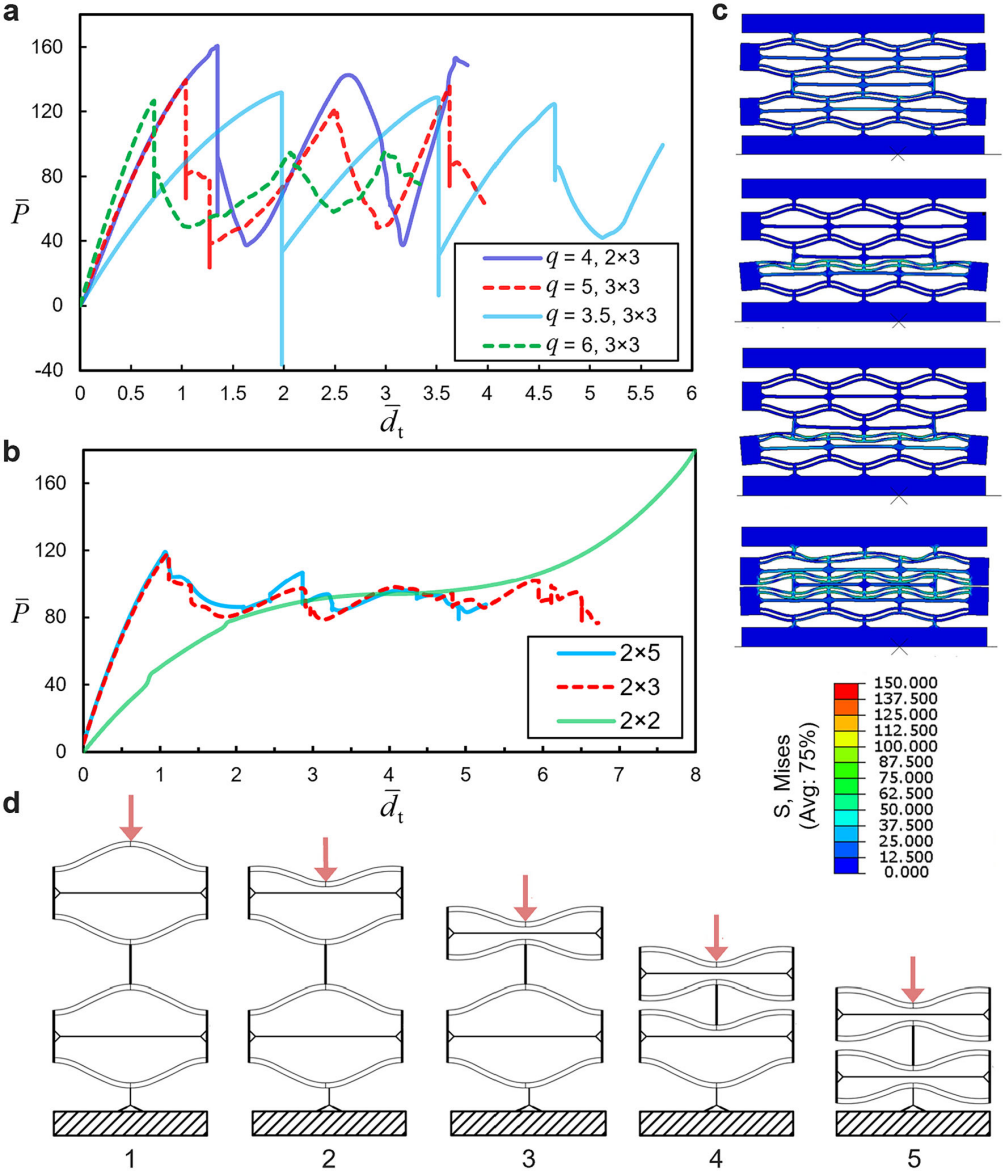
**a** Displacement-controlled response of models with different values of *q*. **b** Force–displacement plots for the model with $q=2$. **c** FEA simulation of the buckling sequence of CDBs in the lattice. **d** Schematics of the typical buckling response of an NSH module

In Fig. 2, NSHM lattices composed of different numbers of cells are compared. As observed, except for small discrepancies following the first peak, the force–displacement plots of NSHM lattices with varying cell sizes virtually converge into a single curve. This indicates that the mechanical response is independent of the lattice size. In Fig. 4a–b, the force–displacement curves of the 3 × 3 lattices are compared. All these models underwent a snap-back mechanism with an average load reduction of 50% following the first peak. The higher the bistability ratio, the lower the maximum compression of the lattice.

The average captured stress, i.e., ${\sigma }_{\text{avg}}=({\sigma }_{\text{max}}+{\sigma }_{\text{min}})/2$, can be determined as the average of the force magnitudes corresponding to the first peak (local maximum) and the first trough (local minimum), respectively, over the CDB cross-sectional area of 4 mm^2^. Notably, ${\sigma }_{\text{avg}}$ decreases with an increase in the bistability ratio, reducing by 16.4% from 24.8 MPa at $q=2$. However, the highest peak stress (42 MPa) was associated with $q=4$, which also exhibited the highest difference between the first local crest and the first trough (${\overline{P} }_{\text{mean}}=135.87$), resulting in a 260% increase (see Fig. 4a,b and 10 of Appendix A).

The force–displacement landscape of the NSHM lattice is characterized by three regimes as follows. Regime I corresponds to the initiation of loading, during which the force increases nonlinearly with the prescribed displacement. In this phase, incipient lateral mid-point deformations in the CDB are observed in the ${\overline{P} }_{1}$ curve, while ${\overline{P} }_{2}$ and ${\overline{P} }_{3}$ are absent in the $\overline{P }$−$\overline{d }$ plot. The beams retain their stability until the load reaches a peak value of ${f}_{1}$. Subsequently, the mode transition occurs, and the beams undergo snap-buckling, characterizing Regime II, which shows a negative tangent slope in the force–deflection plot. The force ${\overline{P} }_{1}$ transitions to either ${\overline{P} }_{2}$ or ${\overline{P} }_{3}$, with a sudden decrease in load due to the snap-back of the CDBs in the lower layers. Since the configuration of the CDB during Regime II is unstable, a sudden transformation to Regime III occurs, marked by the transition from mode 1 to mode 3 ([62]).

Furthermore, as illustrated in Fig. 4, the force–displacement plots of the lattice with $q=2$ exhibited a phase transition only when the number of columns in the lattice exceeded two, i.e., when the lattice was constructed with more than two columns of constituent elements. This may be attributed to the instability of the structure with a small number of constituent elements, flexible beams, and comparatively rigid walls. The actual force–displacement curve of the bistable beam is therefore a hybrid one, where the progression of the force ${\overline{P} }_{1}$ and its transitions are interspersed with either ${\overline{P} }_{2}$ or ${\overline{P} }_{3}$. These transitions occur when ${\overline{P} }_{1}$ intersects with either of the latter forces in the force–displacement landscape (in Regime II, where the lattice manifests negative stiffness). This process continues as the CDBs continue to buckle layer by layer until either the full compression of the lattice is reached, or the lattice recovers elastically as the external energy is converted into internal elastic strain energy within the lattice, without yielding in the elements. Notably, the lattices demonstrate high recoverability compared to conventional honeycomb materials. Under displacement-controlled conditions, the CDBs undergo a snap-through, which causes the structure to experience hysteresis during loading and unloading, leading to energy entrapment within the material.

It should, however, be noted that the buckling sequence is not necessarily in a top-down order and depends on the loading type and conditions, as well as the topology of the lattice. While the buckling process of the NSHM lattice, as discussed in [61, 69] and visualized in Fig. 4d, is assumed to occur in order from the external (top) layers to the internal (bottom) ones, such a scenario seldom occurs under static loading. In the case of lattices with periodic boundary conditions (PBC), the inverted CDBs of the top layer buckle first, followed by the top CDBs of the cells beneath. When no PBC was applied, in the case of a lattice with two layers, the buckling order was reversed, with the top CDBs of the second layer buckling first. However, the buckling of the most internal CDBs generally occurred last.

### Impact loading on the NSHM lattice structure

Dynamic loads of considerable duration are known to cause more significant damage to structures compared to static loads. Dynamic pulse loads can degrade material properties in structural elements that might otherwise withstand static loads of the same magnitude. This is primarily due to the dynamic load factor (DLF), also known as the dynamic amplification factor (DAF), which represents the norm of the complex frequency response function for periodic loads. The DLF is attributed to inertial effects within the structure and the nature of dynamic loads, which induce both initial and residual vibrations. These dynamic loads can arise from various sources such as earthquakes, impacts, blasts, and harmonic or non-harmonic excitations, each potentially driving the structure toward rupture, fatigue, or resonance.

While some solutions have utilized various types of periodic [70–76], modular [77–79], and bio-inspired [80–86] structures as sacrificial layers [87–91] or vibration absorbers [92–95], research has shown that latticed metamaterials can offer effective structural protection against extreme loads such as impact loading [96–99]. Here, we extend the investigation of the dynamic impact response of the NSHM lattice, following the theoretical analyses conducted by the authors and their peers [48, 59, 62]. The models in this work were similar to those examined by the previous authors, set up to be subjected to the impact of a bullet-shaped striker with an initial velocity of ${V}_{0}=5 \text{ m}/\text{s}$ and a mass of $G=5 \text{ kg}$. The formulation of the total potential energy in the dynamic case is similar to that of the static case, except that in the dynamic case, the contribution of kinetic energy to the total internal energy of the structure must be accounted for. To this end, we formulate the kinetic energy term for the ${i}^{\text{th}}$ beam as [62]:

13 $$T_{i}=\frac{h^2b\rho_{b}}{2}\int_{A_0} {\dot{\overline{w}}}_{i}^2{\rm d}A_{{0}},$$

where $d{A}_{0}={\tau }_{\text{b}}d{s}_{0}$ is the differential element of the beam area, and ${\rho }_{\text{b}}$ and $b$ represent the material’s density and width of the beam, respectively. The overdot denotes differentiation with respect to time, as is customary. The equations of motion can be derived by substituting ${\Pi }_{i}$ into the following Euler–Lagrange equation:

14 $$\frac{\partial {\Pi }_{i}}{\partial {A}_{i,j}}-\frac{d}{dt}\left(\frac{\partial {\Pi }_{i}}{\stackrel{.}{\partial {A}_{i,j}}}\right)=0 ,$$

where ${\Pi }_{i}={\overline{U} }_{\text{t},i}$ is the total potential energy, and the index $i$ represents the ${i}^{\text{th}}$ beam. Applying of Eq. 14 to the multi-mode response of the lattice structure yields $n$ ordinary differential equations (ODEs) with respect to each mode $j=\text{1,2}, ...,n$. This is achieved by substituting Eqs. 9 and A2 into Eq. 14. The resulting ODEs involve cross terms corresponding to the buckling modes and alongside linear combination of the inertia terms of the ${j}^{\text{th}}$ mode, as described in Appendix A. The initial kinematic condition of the beam prescribes the initial conditions on the displacement field as $\overline{w }(x,t=0)=0 \,\,$ and $\,\,\dot{\overline{w} }\left(x,t=0\right)=\text{const}.$, while the second condition is satisfied using the conservation of total linear momentum before and after the impact. The expression for the beam momentum is given as:

15 $$M={\int }_{0}^{{l}_{0}}{\rho }_{\text{b}}b\frac{\partial }{\partial t}w\left(x,t\right)d{s}_{0}.$$

Assuming that mode 2 is constrained and higher modes can be neglected, Eq. 15 for the single degree of freedom model leads to, ${\dot{A}}_{1}(0)=\overline{G}\hspace{0.05cm}\overline{{V }_{0}}/(\overline{m }-2\overline{G })$, with $\overline{m }$ denoting the ratio of the mass of the beam to that of the structure.

The impact response of the lattice is governed by three main phases, as follows:

#### Phase 1 (incipient phase)

**Fig. 5:**
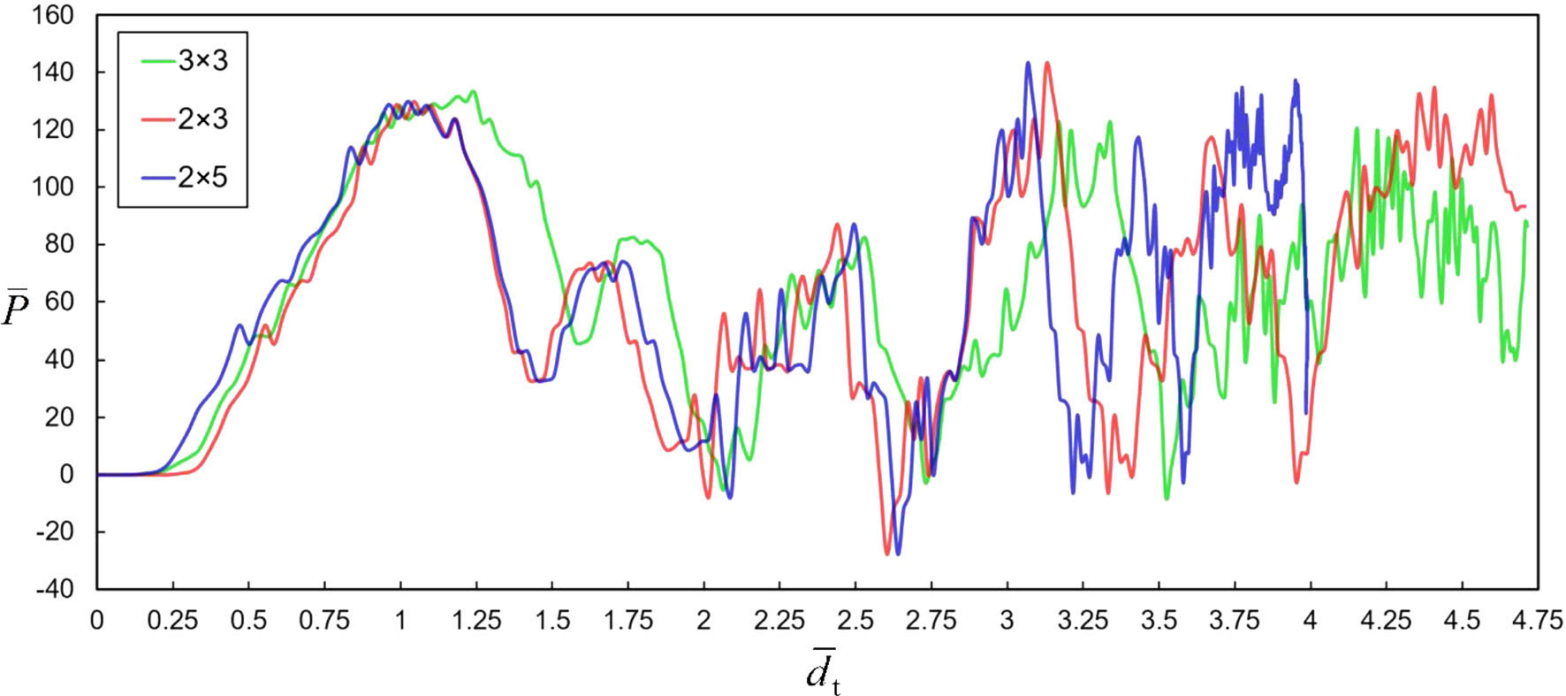
Convergence of force–displacement curves for lattice with $q=4$ subject to the striker impact

This phase triggers the onset of vibration in the top CDBs of the uppermost layer of the lattice (see Fig. 3 and 5). Following the initial vibration of the CDBs, snap buckling occurs due to instabilities in the CDBs.

#### Phase 2 (progressing phase)

During this phase, the transfer of impact energy through dilatational waves to the CDBs in the lower layers triggers the subsequent buckling of the curved beams in these layers.

#### Phase 3 (residual vibration phase)

In this phase, as the striker induces sequential vibrations in the curved beams, its kinetic energy and momentum are transferred to the lattice. This continues until all of the striker’s kinetic energy is converted into strain energy within the lattice structure. Lattice structures that are resilient enough to avoid total compression or densification due to impact repel the striker and recover from the impact. However, the CDBs undergo residual vibrations of smaller amplitude compared to the initial peak, persisting even after the striker has fully detached from the lattice. These residual vibrations, caused by the remaining kinetic energy in the system, eventually dissipate due to structural and other damping mechanisms present in any real system.

The landscape of force–displacement curves, therefore, entails numerous fluctuations throughout the phases of motion (see Fig. 5). As opposed to the static case, the reaction force $\overline{P }$ in dynamic impact loading attains negative values at certain points in time throughout the motion, leading to an energy entrapment mechanism within the structure. In the static case, the attainment of negative values in the force–displacement trajectory depends on Eq. 10 having two real solutions (in addition to the neutral stability point with $d=0$), which is associated with a certain threshold for the apex height. This condition gives rise to two salient features: (i) energy entrapment occurring between two points of zero force, and (ii) a zone of negative energy, both of which are relevant to the static and dynamic cases.

**Fig. 6:**
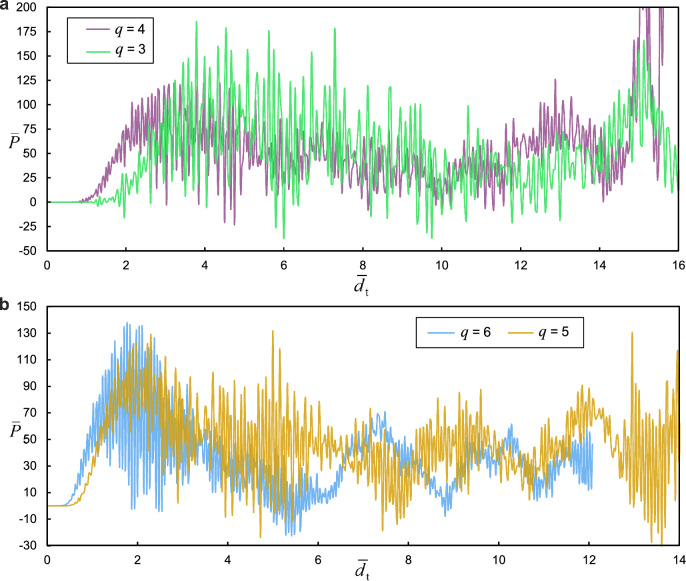
Force–displacement plots of bi-material composites with: **a** *q* = 4 and 3; **b** *q* = 6 and 5

Localized stress bands develop at the midspan of the contact interface between the striker and the lattice. Although the striker impact induces a localized response that depends on the lattice size, as shown in Fig. 6, the plots of the impact force–displacement curves for lattices with different numbers of cells follow similar paths.

In the case of static loading, it turns out that, regardless of the buckling sequence, the final state of the structure will always be as illustrated in part 5 of Fig. 4d, with the total energy absorbed by the structure given by ${E}_{\rm{t}}={\sum }_{i=1}^{n}{E}_{i}$, where ${E}_{i}={\int }_{0}^{{d}_{i}}P(x)dx$. This is not the case when the state of stress reaches the yield point, where energy is expended plastically in the components of the lattice, or in dynamic loading scenarios such as impact or harmonic excitation, where inertia significantly contributes to the stress distribution.

**Fig. 7:**
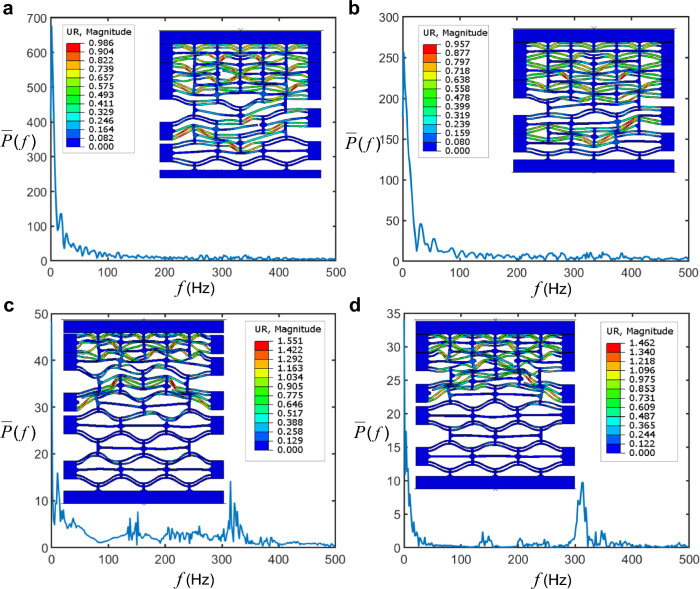
Fast Fourier transform (FFT) of the bi-material composite force–time history: **a** *q* = 4. **b** *q* = 3. **c** *q* = 5. **d** *q* = 6

In dynamic impact scenarios, unlike in static loading, both the mass and stiffness of the beams in the upper layers (or those that buckle first) contribute to the overall strain energy of the beams that have yet to buckle. The mass matrix incorporates the modal mass contribution of each beam. This is also evident from the momentum conservation expression discussed earlier and the kinetic energy expression of the lattice (Fig. 7).

## Impact loading of the bi-material NSHM composite

The analyses above were primarily carried out under static loading conditions, while little work exists in the literature on the response of multi-material composite metastructures to dynamic loads. To address this gap, we examined the performance of a bi-material composite with a homogenized microstructure at the fiber diameter length scale, as examined earlier. These comprised five layers with three constituent elements in each layer that were subjected to uniform plate impact to gain a better understanding of the order of fracture and mechanism of the components within the microstructure. The setup of the bi-material composite impact was analogous to that of [62], except that instead of the capsule-shaped impactor, the plate to induce uniform impact was modeled as a discrete rigid element, prescribed with an initial velocity of 15 m/s, and had a mass of 10 kg. The impactor was constrained in all DOFs except the direction of motion. The average element size of the bi-material composite was 0.5, identical to the static case.

The bi-material construction used PA11 for the CDBs and vertical struts, while the horizontal connectors were made of HIPS material. The latter is a thermoplastic polymer with higher impact resistance and durability than polystyrene. HIPS is more flexible but less ductile than PA11 and exhibits very low isotropic hardening at low strain rates. According to some previous investigations (see, e.g., [54]), for the HIPS specimens loaded to 2% strain and then unloaded, a thousand-fold increase in the strain rate $\dot{\varepsilon }$ from ${10}^{-3}$ min^−1^ increased the yield stress of the material from 18 MPa to nearly 30 MPa. However, the plastic flow exhibited softening, with its slope decreasing as the strain rates increased. Notably, the slope of the plastic flow softening curve also decreased significantly when the specimens were loaded to 3% strain and then unloaded. While the study here examines the mono- and bi-material NSHM composites at low to moderate impact velocities, the strain rate sensitivity of the materials may be included by implementing a viscoplastic material model, such as the Johnson–Cook (JC) constitutive model [100, 101], with parameters of the base materials experimentally determined.

Since the selected material exhibits isotropy, its dynamic plastic flow can well be described using the (JC) constitutive model [102–107]. The JC model is a multiplicative viscoplasticity model in which the dynamic plastic stress ${\sigma }_{\mathrm{y}}$ depends on the stress hardening, strain rate, and temperature functions, expressed as ${\sigma }_{\mathrm{y}}=(A+B{\varepsilon }^{n})(1+C \text{ln }{\dot{\varepsilon }}^{*})(1-{\theta }^{*m})$. In this equation, parameters $A$, $B$, $C$, $m$, and $n$ are five material constants, ${\dot{\varepsilon }}^{*}$ denotes the quotient of the dynamic strain rate to the reference static strain rate (i.e., ${\dot{\varepsilon }}^{*}=\dot{\varepsilon }/{\dot{\varepsilon }}_{0}$), and ${\theta }^{*}$ represents the homologous temperature.

The first bracket in the JC model corresponds to material hardening, for which parameters *A*, *B*, and *n* can be evaluated using the data from Fig. 1d–e, following the procedure detailed in [100, 108]. Similarly, the parameters corresponding to the strain rate sensitivity, i.e. $C$ and $m$, are evaluated using the plastic flow stress at different strain rates and temperatures, as outlined in [108]. Our investigation of the stress–strain curves at various strain rates found the rate sensitivity of PA11 to be negligible (see Table 1). It is also important to note that thermal softening effects are not considered significant and, thus, are not examined in this study. The HIPS material is assumed to follow a ductile damage model, with the values of stress triaxiality and fracture strains provided in Table 1. Additionally, the degradation of stress from the point of necking to the point of fracture was assumed to be linear.

**Table 1:** Material data for HIPS and PA11

Material	*ρ* (g.cm^−3^)	*E* (MPa)	*ν*	*σ*_y_ (MPa)	*B*_1_ (MPa)	*n*	*C*	*T*_M_ (K)	*m*
PA11	1*.*04	1582	0*.*33	42	42*.*64	0*.*4346	0*.*0049	463	1*.*0
HIPS	1*.*04	1215	0*.*34	11*.*86	${\varepsilon }_{\text{f}}$	*η*	/	${\stackrel{.}{\varepsilon }}_{0}$	/
					0.293	0.3	/	1.0	/

**Fig. 8:**
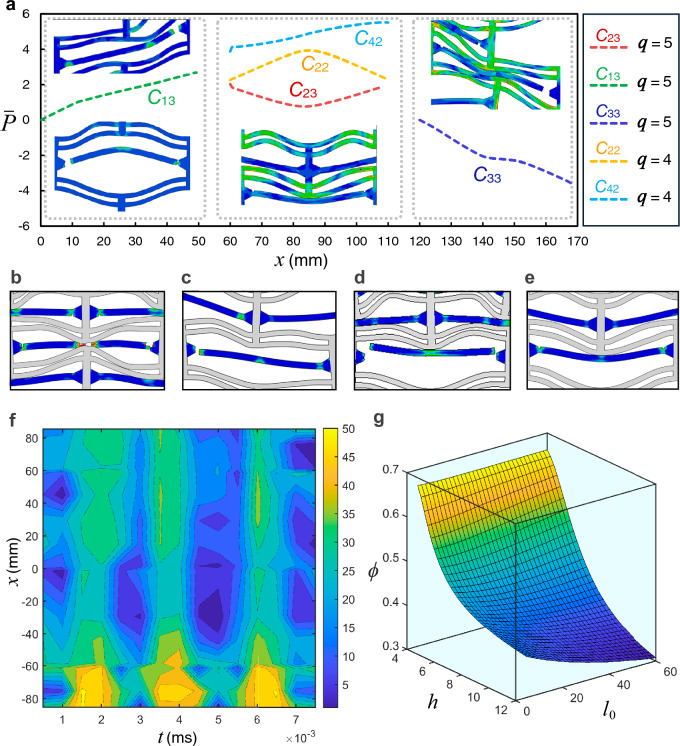
**a** Profiles of the snapped curved beams associated with various cells. **b**-**e** Schematics of fractured elements in the bi-material composite. **f** Mises stress contours across the curved beam for the bi-material composite model with $q=4$. **g** Influence of lattice parameters on relative density

As observed from the results of composite models in Fig. 6, the force–displacement trajectory in response to uniform plate impact is noticeably different from that of the striker impact. In the former case, the response is characterized by higher oscillations, leading to more pronounced peak fluctuations. The force time history curves exhibit greater fluctuations throughout all phases of motion compared to the localized impact case. The force–time histories in the bi-material (analogous to propagated acoustic signals) can be mapped into the frequency domain, as shown in Fig. 8. The sampling frequency was 1000 Hz over the entire duration of the bi-material response.

Although the time histories display several oscillations, the major frequency peaks in the frequency domain are below 150 Hz, while noise and vibrations at higher frequencies are negligible. This indicates that there are no significant high-frequency oscillations in the lattice, which might have otherwise arisen from numerical artifacts or other factors. It is also important to note that while Fourier analysis provides information about the frequency content of the signal or wave, it does not offer time localization, which is provided by wavelet analysis. Wavelets deliver both time and frequency localization, allowing for the analysis of signals at different scales and positions. While this is an intriguing aspect, investigating localized wavelet oscillations requires wavelet transforms, which is beyond the scope of this research.

The onset of deformation in the bi-material composite lattice is accompanied by energy absorption within the lattice CDBs and struts. As deformation progresses, energy is expended plastically in the horizontal connectors, with stress concentrating near their joint connections. Inevitably, the progressive failure and damage of the horizontal connectors increase the average ratio of plastic energy dissipation to elastic energy storage, with the latter being the only retrievable component. Consequently, the recoverability of the composite is noticeably reduced. This ratio, denoted as *η*, typically decreases in models with an increasing bistability ratio. For example, the ratio of energy absorbed elastically to the energy dissipated plastically increased from 9.4% for $q=2$ to $13.1\%$ for $q=6$. When the initial impact velocity increased to *V*_0_ = 15 m/s, all composite models experienced full compression. However, the time taken for full densification was prolonged in lattices with higher bistability ratios.

Following contact between the rigid plate and the bi-material composite, the buckling of the CDBs begins in the top layer and sequentially progresses to the layers beneath. The transition of the CDB buckling mode from mode 1 to mode 3 and back to an inverted mode 1 along the equilibrium path causes the vertical struts to shift relative to the midspan of the flexible horizontal element. This results in the rotation of the latter about the joints and potential debonding from the joints. Such a damage mode was observed as the sole mode in the composites, except in the model with $q=4$, where a couple of horizontal members also exhibited transverse crushing due to compressive contact stresses between two pairs of CDBs on the top and bottom sides of the crushed element (see Fig. 8). In this element, damage initially occurs as the material in the midspan detaches from the horizontal strut surface, followed by outward progression of the damage and simultaneous debonding from the joint. The horizontal elements of the top two layers in the model with $q=5$ do not undergo any fracture despite their rotations. The first snapped elements from their joints pertain to the cells ${C}_{22}$, followed by the detachment of the horizontal beams in cells ${C}_{31}$ and ${C}_{21}$. Detachment of the horizontal connectors from the joints propagates to the layers underneath until full compression of the lattice occurs. Interestingly, the snapping of the horizontal elements in the lower layers does not precede the fracture of all the horizontal elements in the upper layers. The metastructure exhibited larger energy absorption when the bi-materials were acting in tandem as opposed to the metamaterials made of either base material individually. For stiffer structures (with higher $q$), the majority of the impact load is absorbed by the top layers, resulting in higher fluctuations in the force time history before reaching total compression, which corresponds to the average total transverse deformation of the top two layers. Consequently, both the peak load and frequency of vibration sharply settle to residual values around the zero-force point beyond this stage (see Fig. 6(b)). In contrast, a significant portion of the load is transferred to the lower layers.

## Optimization of the NSHM lattice

As discussed previously, the curved beam displacement components in the various examined models interspersed with those of the other beams through their axial deformation. These curved beams exhibit only one stable configuration when $q<2.31$, and two stable configurations (bistability) when $q\ge 2.31$ and mode 2 is constrained. For $q>2.31$, a third solution for $\overline{P }$ (i.e., ${\overline{P} }_{3}$) can exist, provided that mode 2 is constrained. The solution for ${\overline{P} }_{1}$ becomes tangential to that of ${\overline{P} }_{3}$ for $q=2.31$, as demonstrated by [55, 69]. Upon actuation by a transverse load, the CDBs of the latter configuration transition from one equilibrium state to another, corresponding to a phase transformation mechanism.

We employ the MATLAB Toolbox *fmincon* as an embedded optimization tool, implementing the Euler–Lagrange expression. This toolbox is a built-in local minimization function that determines a vector variable $\mathbf{x}$ such that the multivariable function $f(\mathbf{x})\ge 0$ is minimized. In this case, the function to minimize is the total potential energy of the lattice structure, which is the sum of the total energies of the CDBs, assuming the horizontal and vertical struts experience only rigid motion. This can be achieved by increasing the central strut wall thickness. The variable of interest for optimization is a vector whose components correspond to the modal amplitudes ${A}_{j,i}$ ​of the CDB, with $i$ representing the beam number and $j$ corresponding to the deformation mode. It is important to note that $\text{mode }n$ refers to the term containing $n$ in the argument of the function used in the approximating expansion of the displacement field.

**Fig. 9:**
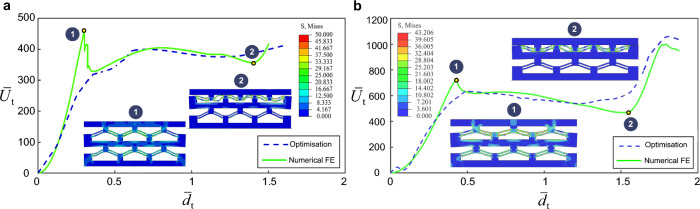
Finite element numerical validation of the optimization results with **a** $q=4$ and **b** $q=5$

The non-dimensional displacement vector corresponding to each column of the constituent elements is given as $({\overline{d} }_{1},{\overline{d} }_{2},{\overline{d} }_{3}, ...),$ with $0\le {\overline{d} }_{i}\le 2$, while the strain energy components of each CDB in the constituent element are $({\overline{U} }_{1}({\overline{d} }_{1}),{\overline{U} }_{2}({\overline{d} }_{2}),{\overline{U} }_{3}({\overline{d} }_{3}), ...),$ respectively. The objective function is the total potential energy of the system ${\overline{U} }_{\mathrm{t}}={\sum }_{i=1}^{n}{\overline{U} }_{i}$, which is to be minimized with respect to modal deformations ${A}_{j}$. The algorithm enforces a linear constraint of ${\overline{d} }_{n}={\overline{d} }_{\text{t}}-{\sum }_{i=1}^{n-1}{\overline{d} }_{i}$ (where ${\overline{d} }_{\mathrm{t}}$ is the total transverse displacement of the lattice) and a series of nonlinear constraints enforced by the Euler–Lagrange equations, i.e., $\partial {\overline{U} }_{\mathrm{m}}/\partial {A}_{j}+\partial {\overline{U} }_{\mathrm{b}}/\partial {A}_{j}-\partial {\overline{W}}_{\mathrm{t}}/\partial {A}_{j}=0$ as a nonlinear optimization, with *W* denoting the work done on the lattice. The optimization algorithm, expressed as follows, is then implemented, and the equations are solved in MATLAB in the sequel. In Fig. 9, we compare the contour plots of the strain energy of the homogenized lattice and the total displacement of the NSHM lattices for different bistability ratios.

16 $$\begin{aligned}&{\text{min }}\bar{U}_{{\text{t}}} = \sum\limits_{{i = 1}}^{n} {\bar{U}_{i} }\\ &{\textrm{with respect to }} \left({\overline{d} }_{1},{\overline{d}}_{2},{\overline{d}}_{3},\dots \right)\\ &{\text{subject to }}\bar{d}_{n} = \bar{d}_{{\text{t}}} - \sum\limits_{{i = 1}}^{{n - 1}} {\bar{d}_{i} {\text{ and }}\frac{{\partial \bar{U}_{{\text{t}}} }}{{\partial A_{j} }} = 0}\end{aligned}$$

Using the algorithm described above, the fixed value of ${\overline{d} }_{\text{t}}$, which depends on the geometry of the lattice, can be discretized at every step of the motion. To this end, we define a time increment ${\delta t}$ with a fixed value of ${\overline{d} }_{\text{t}}$, based on the maximum compression of the NSHM lattice with two rows and three columns (see Fig. 9).

To effectively constrain the rotational degrees of freedom and thereby limit undesirable modes—achieving homogeneous deformations throughout the lattice—geometry optimization is required. The geometry of the NSHM is governed by an interplay between the bistability ratio, horizontal stiffeners, and wall thicknesses, all of which affect the microstructure’s porosity. Following Eqs. (2) and (3), as illustrated in Fig. 2(c) and Fig. 3(b,c), an increase in the bistability ratio results in a more porous microstructure. This, in turn, leads to greater recoverability and energy absorption, compared to the energy dissipated plastically throughout the microstructure. However, to achieve homogeneous deformations and eliminate relative deformations or rotational degrees of freedom, the increase in porosity from a higher bistability ratio must be counterbalanced by an increase in cell wall thickness and height. For design purposes, an ideal microstructure may be fabricated with a cell wall thickness at least five times greater than the curved beam thickness and constructed from a rigid material such as PLA. At the same time, a high bistability ratio ($q>4$) should be targeted, with the CDBs made from softer polymers to ensure flexibility.

## Concluding remarks

In this work, we presented an extensive analytical and numerical analysis of negative-stiffness multistable bi-material composites, consisting of a periodic arrangement of curved double beams (CDBs). The constituent curved beams in the lattice exhibited bistability, meaning they had two stable positions and could transition from one stable equilibrium state to the other. A theoretical model was developed, in line with the authors’ previous research, to derive a solution for the absorbed strain energy of a lattice made up of two periodic cells using a nonlinear optimization technique. The bistability of the curved beams was induced by an increase in the bistability ratio parameter which also resulted in lower permanent deformation and enhanced lattice stiffness. The influence of the plunger width and the gap between the beams was also examined. The results indicated that the relative displacements between the two beams in a CDB pair could only be minimized by increasing the number of connectors at various positions on the CDBs. In contrast, increasing the center plunger or the gap between the beams did not mitigate the non-synchronous motion of the curved beams. However, asynchronous motion of the curved beams was reduced in CDBs with higher bistability ratios.

For lattices made of three or more columns of constituent elements, the response of the lattice was found to be independent of the number of cells, as the force–displacement histories of the lattices virtually converged to a single curve. Furthermore, although a 2 × 2 lattice with $q=2$ exhibited an unstable response with no negative stiffness phenomenon occurring throughout the motion, a slight negative slope in the force–deflection curve was observed when a higher number of cells was used.

The analyses of the striker impact and plate impact loading on the lattice, fabricated from single PA and bi-materials of PA-HIPS in composite action, were conducted within the context of the problem. In the former case, the overall force–displacement plots of the lattice were relatively independent of the number of cell columns. Lattice structures with lower bistability ratios exhibited more fluctuations in the force–time history than the stiffer ones with higher bistability ratios, consistent with previous findings [62]. Furthermore, the residual vibration of the lattice around the zero-force line, following the detachment of the striker from the metastructure and full recovery, decreased with an increase in $q$.

In the latter case, the composite lattice response showed energy absorption and wave propagation limitation within the top layers as the bistability ratio increased. In contrast, the propagation of dilatational waves throughout the layers of lattices with lower bistability ratios led to significant compression of the structure, as well as progressive failure of the horizontal struts. The total energy dissipated plastically decreased by 4% as the bistability ratio increased from $q=2$ to $q=6$.

While the dimensionless first peak force of the lattice with $q=6$ (also the maximum in the range of $0\le {\overline{d} }_{\text{t}}\le 12$) was slightly higher than that of $q=5$ (138 as opposed to 131), its maximum transverse displacement exceeded by 20% (rising from 12 for $q=4$ to 14.5 for $q=6$), and by 50% when considering the range of $q=3$ to $q=6$. In the vicinity of fluctuations ($2.3\le {\overline{d} }_{\mathrm{t}}\le 4.2$) following the first peak, the average force decreased by 77% when transitioning from $q=3$ to $q=6$, and by approximately 27% in the transition from $q=5$ to $q=6$. As a result, lattice structures with relatively high bistability ratios are stiffer and have a greater capacity for impact energy absorption, making them preferable in industrial applications where energy absorption and high stiffness are critical. A progressive collapse of the horizontal elements was also observed in lattices with low CDB apex heights. However, the failure of horizontal struts typically initiated in the elements on the left and right sides of the lattice, followed by those in the middle.

As a final remark, it should be noted that in practical applications, using very high values of bistability ratio to achieve better stiffness and resilience would also increase the overall depth of the structure and may not be a viable solution, depending on the cell sizes and application. Thus, the microstructure of phase-transforming lattices will require shape and topology optimizations to strike a balance between the apex height, wall thickness, the distance between the top surface and the curved beams, and the gap between the CDB and the horizontal struts. These topics merit further investigation, which could be the subject of a follow-up study.

## A Appendix

### A.1: Static response of the CDB bistable structure

**Fig. 10:**
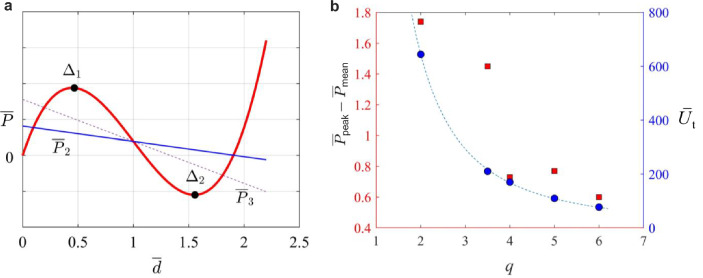
**a** Three kinds of solutions to Eq. 9. **b** Variations of force amplitudes around their average values and the total strain energy of the lattice with bistability ratio

When considering modes 1, 2, and 3 only, the equilibrium equation for the total potential variation of a curved beam, $\partial \overline{U }\ge 0$ (or $\partial \overline{U }/\partial {A}_{j}=0$), leads to three expressions as follows (Fig. 10)

A1a $$\textrm{ODE} 1:12{{q}}^{2}\left(\frac{1}{16}{{{\omega}}}_{1}^{2}-\frac{1}{4}\sum\limits_{{{j}}=1}^{3}{{{A}}}_{{{j}}}^{2}{{{\omega}}}_{{{j}}}^{2}\right){{{A}}}_{1}{{{\omega}}}_{1}^{2}+\left(\frac{1}{2}-{{{A}}}_{1}\right){{{\omega}}}_{1}^{4}-4\overline{{{P}} }=0$$

A1b $$\mathrm{ODE}2:{A}_{2}{\omega}_{2}^{2}\left(\left(\left({{{A}}}_{1}^{2}+4{{{A}}}_{3}^{2}-\frac{1}{4}\right){{{\omega}}}_{1}^{2}+{{{A}}}_{2}^{2}{{{\omega}}}_{2}^{2}\right){{{q}}}^{2}+\frac{1}{3}{{{\omega}}}_{2}^{2}\right)=0$$

A1c $$\mathrm{ODE}3:{A}_{3}{{{\omega}}}_{3}^{2}\left(\left({{{A}}}_{3}^{2}{{{\omega}}}_{3}^{2}+\left({{{A}}}_{1}^{2}-\frac{1}{4}\right){{{\omega}}}_{1}^{2}+{{{{A}}}_{2}^{2}{{\omega}}}_{2}^{2}\right){{{q}}}^{2}+\frac{1}{3}{{{\omega}}}_{3}^{2}\right)=0$$

### A.2: Dynamic impact response of the CDB bistable structure

Substituting Eq. 5 into Eq. 13, we obtain

A2 $${{{T}}}_{{{i}}}=\frac{{{{h}}}^{2}{{{m}}}_{}}{2{{{s}}}_{0}}{\int }_{0}^{{{{l}}}_{0}}\left(\sum\limits_{{{j}}=1}^{{{n}}}\sum\limits_{{{k}}=1}^{{{m}}}{\stackrel{.}{{{A}}}}_{{{j}}}{\stackrel{.}{{{A}}}}_{{{k}}}{{{W}}}_{{{j}}}\left({{X}}\right){{{W}}}_{{{k}}}({{X}})\right)\mathrm{{d}}{{{s}}}_{0},$$

where ${m}_{\text{b}}={\rho }_{\text{b}}\tau b{s}_{0}$ represents the beam mass, and ${\rho }_{\text{b}}$, $\tau$, and $b$ denote the beam density, depth, and width, respectively. The initial length of the beam is expressed as

A3 $$\mathrm{d}{{{s}}}_{0}\approx \left(1+\frac{{{{\pi}}}^{2}{{{h}}}^{2}}{2{{{l}}}_{0}^{2}}{\mathrm{sin}}^{2}\left(2{{\pi}}{{X}}\right)\right)\mathrm{d}{{X}}.$$

Substituting Eq. A2 into the second part of Eq. 14, each resultant ODE will include an inertia component of mode $i$ as ${\ddot{A}}_{i}$, alongside the inertia components of the other modes, expressed as

A4 $$\frac{\mathrm{d}}{\mathrm{d}{t}}\left(\frac{\partial {{{\Pi}}}_{{{i}}}}{\partial {\stackrel{.}{{{A}}}}_{{{i}}}{ }}\right)=\frac{6{{{l}}}_{0}^{3}{{{\rho}}}_{\mathrm{b}}}{{{E}}{{{\tau}}}_{\mathrm{b}}^{2}}{\int }_{0}^{{{{l}}}_{0}}\sum\limits_{{{k}}=1}^{{{m}}}{\stackrel{..}{{{A}}}}_{{{k}}}{{{W}}}_{{{i}}}\left({{X}}\right){{{W}}}_{{{k}}}({{X}})\mathrm{d}{{{s}}}_{0}.$$

The multi-mode ODEs can be solved using the finite difference method or MATLAB’s ODE45 solver.

## Data Availability

Data will be made available upon reasonable request.

## Latin

${A}_{0}$: Area of the beam
${A}_{\text{a}}$: Unit cell aperture area
${A}_{\text{f}}$: Unit cell infill area
${A}_{j}$: *j*^th^ modal amplitudes of the displacement field
$C$: Johnson Cook strain rate parameter
$$E$$: Young’s modulus
$$G$$: Striker mass
$I$: Second moment of area
${L}_{1}$: Length of unit cell in the *x*-direction
${L}_{2}$: Length of unit cell in the *y*-direction
$$M$$: Momentum
$$P$$: Actuation force
${P}_{i}$: ${i}^{\text{th}}$ Kind of solution to the actuation force
${\overline{P} }_{\text{peak}}$: Dimensionless peak nodal force
${\overline{P} }_{\text{mean}}$: Dimensionless mean nodal force
${S}_{0}$: Dimensionless initial length of the beam
${T}_{i}$: Kinetic energy of the ${i}^{\text{th}}$ curved beam
$$U$$: Energy
$${U}_{\text{b}}$$: Bending energy
$${U}_{\text{m}}$$: Membrane energy
${U}_{\text{t}}$: Total potential energy of the lattice
${V}_{0}$: Striker initial velocity
${W}_{j}$: Curved beam modal shapes of the *j*^th^ mode
$$X$$: Dimensionless *x*-coordinate
$b$: Curved beam out-of-plane thickness
$d$: Transverse displacement of the curved beam
${d}_{i}$: Transverse displacement of the *i*^th^ cell
${\overline{d} }_{\text{t}}$: Dimensionless total transverse displacement of the lattice
$h$: Apex height of the curved beam
${h}_{\text{w}}$: Height of the cell wall
${l}_{0}$: Midspan of the curved beam
${m}_{\text{b}}$: Mass of the curved beam
$m$: Mass of the lattice
$$p$$: Membrane force
$$q$$: Bistability ratio
${s}_{0}$: Initial length of the curved beam
${w}_{j}$: Transverse displacement of the *j*^th^ mode

## Greek

${\Pi }_{i}$: Total potential energy function of the *i*^th^ beam
${\tau }_{\text{b}}$: Curved beam thickness
${\tau }_{\text{hb}}$: Thickness of the horizontal beams
${\tau }_{\text{cm}}$: Horizontal width of the cell wall wedge
${\tau }_{\text{g}}$: CDB gap width
${\tau }_{\text{w}}$: Vertical connector strut thickness
$$\nu$$: Poisson’s ratio
$$\rho$$: Density
${\rho }_{\text{b}}$: Curved beam density
$$\sigma$$: Stress tensor
$${\sigma }_{\mathrm{y}}$$: Yield strength
$${\sigma }_{\text{avg}}$$: Average stress
${\omega }_{j}$: Modal frequency of the *j*^th^ mode
